# A new proposal for phenotypic classification and outcome assessment of dermatomyositis based on clinical manifestations and serological testing^⋆^

**DOI:** 10.1016/j.abd.2023.06.005

**Published:** 2024-03-23

**Authors:** Ting Huang, Ting Ding, Liqing Ding, Shasha Xie, Xiaojing Li, Qiming Meng, Xiaomeng Wu, Hui Luo, Hongjun Zhao

**Affiliations:** a Department of Rheumatology and Immunology, Xiangya Hospital of Central South University, Changsha, China; b Provincial Clinical Research Center for Rheumatic and Immunologic Diseases, Xiangya Hospital, Changsha, China; c National Clinical Research Center for Geriatric Disorders, Xiangya Hospital, Changsha, China

**Keywords:** Classification, Cohort studies, Dermatomyositis, Health care, Interstitial lung disease, Outcome assessment

## Abstract

**Background:**

Dermatomyositis (DM) is an infrequent disease subgroup of idiopathic inflammatory myopathies characterized by distinct skin lesions. However, high heterogeneity makes clinical diagnosis and treatment of DM very challenging.

**Objectives:**

Unsupervised classification in DM patients and analysis of key factors related to clinical outcomes.

**Methods:**

This retrospective study was conducted between 2017 and 2022 at the Department of Rheumatology, Xiangya Hospital, Central South University. 162 DM patients were enrolled for unsupervised hierarchical cluster analysis. In addition, we divided the clinical outcomes of DM patients into four subgroups: withdrawal, stabilization, aggravation, and death, and compared the clinical profiles amongst the subgroups.

**Results:**

Out of 162 DM patients, three clusters were defined. Cluster 1 (n = 40) was mainly grouped by patients with prominent muscular involvement and mild Interstitial Lung Disease (ILD). Cluster 2 (n = 72) grouped patients with skin rash, anti-Melanoma Differentiation Associated protein 5 positive (anti-MDA5^+^), and Rapid Progressive Interstitial Lung Disease (RP-ILD). Cluster 3 (n = 50) grouped patients with the mildest symptoms. The proportion of death increased across the three clusters (cluster 3 < cluster 1 < cluster 2).

**Study limitations:**

The number of cases was limited for the subsequent construction and validation of predictive models. We did not review all skin symptoms or pathological changes in detail.

**Conclusions:**

We reclassified DM into three clusters with different risks for poor outcome based on diverse clinical profiles. Clinical serological testing and cluster analysis are necessary to help clinicians evaluate patients during follow-up and conduct phenotype-based personalized care in DM.

## Introduction

Idiopathic Inflammatory Myopathies (IIMs) are groups of autoimmune diseases of unknown etiology characterized by skeletal muscle involvement, with extensive and diverse extra-muscular involvement.^1^ Dermatomyositis (DM) is a disease subgroup of IIM characterized by distinct skin lesions. Cutaneous manifestations are variable in DM, in particular Gottron papules and heliotrope rashes. Muscular manifestations are presented as symmetric, proximal muscle weakness.^2^ Myositis-Specific Antibodies (MSA) such as anti-complex nucleosome remodeling histone deacetylase (anti-Mi2), anti-Melanoma Differentiation Associated protein 5 (anti-MDA5), anti-Nuclear Matrix Protein-2 (anti-NXP2), anti-Transcription Intermediary actor-1γ (anti-TIF1γ) and anti-SUMO-Activating Enzyme Subunit SAE (anti-SAE) were demonstrated to be highly relevant to the diagnosis and clinical presentation of DM. Comprehensive analysis of clinical manifestations and laboratory tests enable clinicians to effectively assess patients.

Nevertheless, marked heterogeneity makes the management of DM much more challenging, of note, there are still no comprehensive consensus-driven guidelines for DM. Clinically, few patients could obtain drug-free remission,^3^ outcomes were unsatisfactory in most patients, such as recurred cutaneous rash or muscle inflammation, and some of them suffered from Rapidly Progressive Interstitial Lung Disease (RP-ILD) and had high mortality.^4^ Therefore, detailed outcome evaluation or risk factor studies in DM are essential for early assessment.

Exploring new classification systems has the potential to lead to faster disease detection, a more accurate picture of disease status, and rapid response to disease outbreaks. Ideally, subgroups share common characteristics in terms of symptoms, pathogenesis, and prognosis. In this context, this study aims to enhance patient management by proposing a promising classification approach for the diagnosis and prognosis assessment of DM based on clinical serological profiles.

## Methods

### Statement of ethics

The study was approved by the Medical Ethics Committee of Xiangya Hospital of Central South University in Changsha, China. All patients provided written informed consent. The study was conducted in accordance with the Declaration of Helsinki.

### Patients

This was a single-centre retrospective study conducted between 2017 and 2022 in the Department of Rheumatology and Immunology, Xiangya Hospital of Central South University. Demographic information, clinical manifestations, laboratory findings, and clinical outcomes were recorded, only patients with an exhaustive set of data were included for further analysis. Western blotting and 16 items of the anti-myositis spectrum kit (European) were used to detect serum antibodies. The authors included 162 DM patients (new-onset or treated) defined by the criteria of Bohan and Peter.^5^, ^6^ The diagnosis of ILD was based on respiratory symptoms and High-Resolution Computed Tomography (HRCT).^7^ RP-ILD was established if a patient exhibited two or more of the following within 3-months of the onset of ILD: (1) Progressive dyspnea, (2) New Ground Glass Attenuation (GGA) or consolidation on HRCT, and (3) Hypoxemia with >10 mmHg decrease in arterial oxygen tension.^8^

Patients were discriminated between older and younger than 50 years of age, reasons for the separation are as follows: based on epidemiological data from relevant authoritative reviews, the peak age for dermatomyositis is 50 years. Another reason is the need for data type conversion.

Patients in the treatment withdrawal group were no longer treated with any medication. Stabilization was defined as regular glucocorticoid reduction and improved symptoms for longer than a 6-month continuous period. Aggravation was defined as failing glucocorticoid and/or ≥1 immunosuppressive agent treatment, glucocorticoid supplementation or replacement of immunosuppressive agent was required (excluding replacement due to drug side effects). In the death group, causes included acute respiratory failure, multiple organ failure, and cachexia.

### Statistical analysis

Quantitative data (median [inter quartile range]) and qualitative data (frequency and percentage) were described. Unsupervised descriptive methods of statistical learning were used to analyze the cohort. Hierarchical clustering analysis was used to aggregate patients into subgroups, and Euclidean distance and Ward's agglomerative method were used to unsupervisedly cluster patients. Ward minimizes the variance and hierarchy of the data, and when the authors use ward linkage, we only use the Euclidean distance metric. Furthermore, the authors also visualized this data by reducing the dimension using *t*-Distributed Stochastic Neighbor Embedding (tsne), as it uses different distances to represent overlapping points and adopts long-tail distribution, thus solving such “crowding problem”.

In addition, nonparametric tests were used for data with non-normal distributions, and chi-squared tests were used for categorical variables, p-values were adjusted by Bonferroni correction, ≤0.05 was considered significant.

All analyses were performed using MATLAB (version R2021a, MathWorks.Inc) and GraphPad Prism software (version 8).

## Results

### Unsupervised clustering of DM

**Figure 1:**
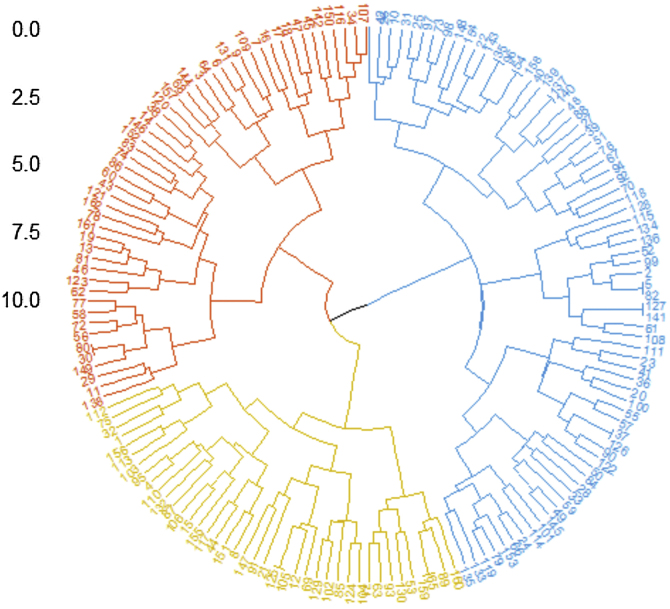
Unsupervised hierarchical analysis tree of DM patients. Hierarchical clustering analysis of 162 DM patients revealed three main clusters (cluster 1: yellow, cluster 2: blue, cluster 3: red). The Euclidean distance and Ward agglomeration methods were used to generate the dendrogram.

**Figure 2:**
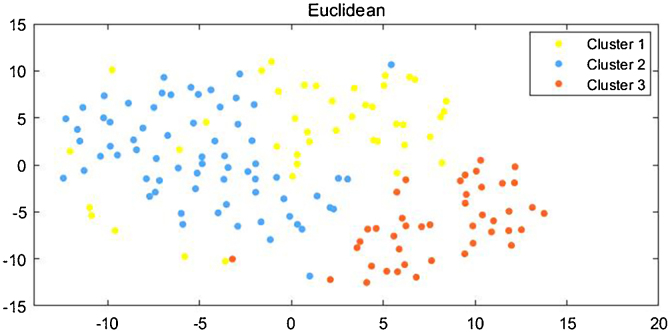
Visualized subgroups of 162 DM patients by tsne algorithm. Scatter plot map showing the individuals and clusters they belonged to in DM. tsne algorithm was used to reduce the dimensionality of the data.

**Figure 3:**
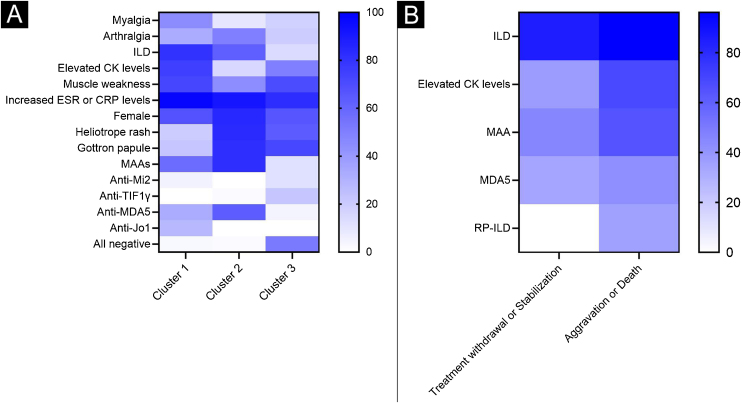
Heatmaps of a wide range of symptoms in different subgroups. The heatmap summarizes the frequency distribution of the main variables across clusters. Color codes indicate the highest frequency in blue (100%), and the lowest frequency in white (0%). (A) Heatmap of the three clusters. (B) Heatmap of groups with different outcomes.

First, the authors enrolled 162 DM patients in this study, whose baseline profiles were recorded at the time of their first hospitalization at Xiangya Hospital, and we also tracked the last outpatient visits of these patients. Among those patients, 121 (74.7%) were female, 53.1% of patients were older than 50 years old, median and Interquartile Range (IQR) of the baseline disease course was 6 (3, 20.5) months, median and IQR of follow-up was 17 (9, 38) months, the longest follow-up course was 136-months. Three clusters within DM patients were identified by the unsupervised hierarchical analysis tree (Fig. 1) and further visualized by tsne (Fig. 2). Characteristics of each cluster were reported in Table 1 (For post-hoc tests, the Bonferroni method was used for p-value adjustment, see supplementary Table S1) and Fig. 3.

**Table 1:** Unsupervised hierarchical analysis: demographics and clinical characteristics.

Variables	Total	Cluster 1	Cluster 2	Cluster 3	p-value
	n = 162	n = 40	n = 72	n = 50	
General information					
Female, n (%)	121 (74.7)	27 (67.5)	61 (84.7)	33 (66.0)	**0.031**
Age (≥50 years), n (%)	86 (53.1)	20 (50.0)	41 (56.9)	25 (50.0)	0.679
Disease course, median (range), months	6 (3, 20.5)	10 (3, 24)	5 (2, 10)	9 (3, 36)	0.070
Follow-up, median (range), months	17 (9, 38)	13 (10, 27.2)	17.5 (6, 39)	20 (12, 38.2)	0.431
Clinical manifestations					
Skin					
Skin ulcer, n (%)	27 (16.7)	6 (15.0)	16 (22.2)	5 (10.0)	0.194
Heliotrope rash, n (%)	100 (61.7)	8 (20.0)	60 (83.3)	32 (64.0)	**<0.001**
Gottron papule, n (%)	104 (64.2)	9 (22.5)	59 (81.9)	36 (72.0)	**<0.001**
Mechanic’s hand, n (%)	12 (7.4)	4 (10.0)	6 (8.3)	2 (4.0)	0.506
Muscle & Joint					
Myalgia, n (%)	34 (21.0)	18 (45.0)	7 (9.7)	9 (18.0)	**<0.001**
Muscle weakness, n (%)	96 (59.3)	29 (72.5)	32 (44.4)	35 (70.0)	**0.003**
Arthritis, n (%)	18 (11.1)	3 (7.5)	12 (16.7)	3 (6.0)	0.176
Arthralgia, n (%)	59 (36.4)	13 (32.5)	36 (50.0)	10 (20.0)	**0.003**
Lung					
ILD, n (%)	84 (51.9)	32 (80.0)	45 (62.5)	7 (14.0)	**<0.001**
RP-ILD, n (%)	27 (16.7)	5 (12.5)	21 (29.2)	1 (2.0)	**<0.001**
Elevated CK levels, n (%)	66 (40.7)	30 (75.0)	11 (15.3)	25 (50.0)	**<0.001**
Increased ESR or CRP levels, n (%)	146 (90.1)	39 (97.5)	66 (91.7)	41 (82.0)	**0.049**
Auto - antibody					
MSAs, n (%)	88 (54.3)	18 (45.0)	49 (68.1)	21 (42.0)	**0.007**
Anti-Mi2, n (%)	8 (4.94)	2 (5.0)	0 (0.0)	6 (12.0)	**0.005**
Anti-TIF1γ, n (%)	12 (7.4)	0 (0.0)	1 (1.4)	11 (22.0)	**<0.001**
Anti-MDA5, n (%)	61 (37.7)	13 (32.5)	46 (63.9)	2 (4.0)	**<0.001**
Anti-NXP2, n (%)	5 (3.1)	3 (7.5)	1 (1.4)	1 (2.0)	0.162
Anti-SAE, n (%)	2 (1.2)	0 (0.0)	1 (1.4)	1 (2.0)	1.000
Anti-Jo1, n (%)	11 (6.8)	11 (27.5)	(0.0)	0 (0.0)	**<0.001**
Anti-PL7, n (%)	8 (4.9)	4 (10.0)	4 (5.6)	0 (0.0)	0.062
Anti-PL12, n (%)	3 (1.9)	1 (2.5)	2 (2.8)	0 (0.0)	0.609
Anti-EJ, n (%)	2 (1.2)	1 (2.5)	1 (1.4)	0 (0.0)	0.724
Anti-OJ, n (%)	0 (0.0)	0 (0.0)	0 (0.0)	0 (0.0)	‒
MAAs, n (%)	88 (54.3)	23 (57.5)	59 (81.9)	6 (12.0)	**<0.001**
All negative, n (%)	28 (17.3)	1 (2.5)	1 (1.4)	26 (52.0)	**<0.001**

Data were given as median (range), or as number and percentage. Elevated CK level were defined as >200 U/L. Increased ESR level were defined as >20 mm/h (female) or >15 mm/h (male). Increased CRP level were defined as >10 mg/L.

ESR, Erythrocyte Sedimentation Rate; CRP, C-Reactive Protein; ILD, Interstitial Lung Disease; RP-ILD, Rapidly Progressive Interstitial Lung Disease; CK, Creatine Kinase; MSAs, Myositis-Specific Antoantibodies; Mi2, Complex nucleosome remodeling histone deacetylase; TIF1γ, Transcription Intermediary Factor-1γ; MDA5, Melanoma Differentiation Associated protein 5; NXP2, Nuclear Matrix Protein-2; SAE, SUMO-Activating Enzyme subunit SAE, Jo1, Histidyl-ARN-t-synthetase; PL7, Threonine-ARN-tsynthetase; PL12, Alanine-ARN-tsynthetase; EJ, glycyl-ARN-t-synthetase; OJ, Isoleucyl-ARN-t-synthetase; MAAs, Myositis-Associated Autoantibodies.

All variables above (excluded “disease course”, “follow-up” and “RP-ILD”) were involved in the hierarchical cluster analysis. For post-hoc tests, Bonferroni method was used for p-value adjustment, see Supplementary Table S1. Bold font for p ≤ 0.05.

Cluster 1 (n = 40, 24.7%) grouped DM patients with dominant muscular involvement and mild ILD. The main symptoms in this cluster included myalgia, muscle weakness, elevated Creatine Kinase (CK) levels, increased Erythrocyte Sedimentation Rate (ESR) or C-Reactive Protein (CRP) levels, a higher proportion of positive anti-histidyl-tRNA synthetase (anti-Jo1) (27.5% in cluster 1 versus 0.0% in cluster 2 or cluster 3), or positive anti-aminoacyl-tRNA Synthetase (ARS) (42.5% in cluster 1 versus 9.7% in cluster 2 or 0.0% in cluster 3). Although ILD was present in 80% of patients, very few of them progressed to RP-ILD.

Cluster 2 (n = 72, 44.4%) grouped patients with dermatomyositis-specific rash, arthralgia, ILD and increased ESR or CRP levels. 63.9% of the patients were anti-MDA5 positive and 81.9% were Anti-myositis-associated autoantibodies (anti-MAAs) positive.

Cluster 3 (n = 50, 30.9%) was the mildest type, with fewer joint symptoms, the lowest rate of ESR, CRP and ILD. Besides, the myositis antibody profile was negative in 52% of patients. 12% of the patients were anti-Mi2 positive (versus 5% in cluster 1 and 0% in cluster 2) and 22% were anti-TIF1γ positive (versus 0% in cluster 1 and 1.4% in cluster 2).

Overall, the clinical phenotypes of the three clusters were significantly different, with cluster 1 having a significant muscle involvement, while cluster 2 was predominantly skin-involved and anti-MDA5 positive, both clusters had high proportions of ILD and inflammatory burden. Cluster 3 was the mildest type.

### Clinical outcomes in the three clusters

**Figure 4:**
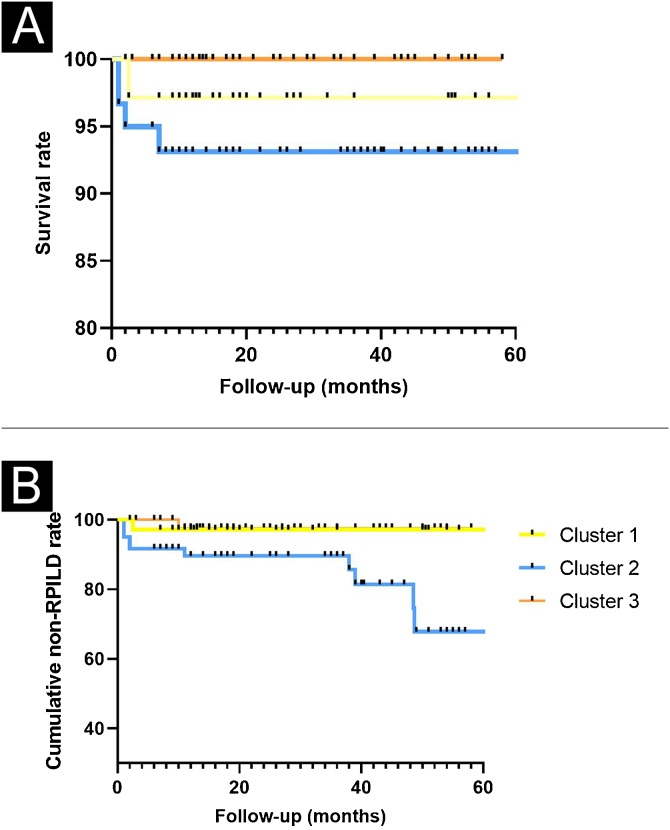
Survival rates and cumulative non-RPILD rates across the three clusters. Compared to the other two clusters, cluster 2 has the lowest cumulative survival rate (A) and non-RPILD rate (B).

**Figure 5:**
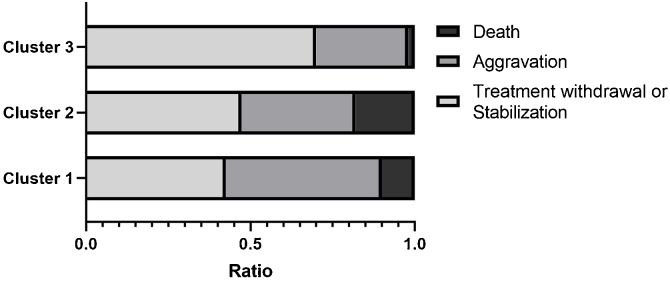
Proportion of different clinical outcomes in the three clusters. The proportion of “Death” increased successively in the three clusters (cluster 3 < cluster 1 < cluster 2).

We then compared the complications and clinical outcomes in these 3 clusters of DM patients. Complications included malignancy, RP-ILD, deterioration of general condition, pulmonary arterial hypertension, and septic shock. The cumulative incidence of non-RPILD was the highest in cluster 3, moderate in cluster 1 and the lowest in cluster 2. During the 60-month follow-up period, patients in cluster 3 had the highest survival, while those in cluster 2 had the lowest survival. In addition, the majority of deaths occurred during the first 3-months of follow-up (Fig. 4). The present data further indicated that the percentages of “RP-ILD” and “Deterioration of general condition” were extremely higher in cluster 2. The proportion of death increased across the three clusters (cluster 3 < cluster 1 < cluster 2) (Fig. 5).

The authors divided the clinical outcomes into four subgroups: treatment withdrawal, stabilization, aggravation, and death. 53.1% of patients maintained their treatment withdrawal or stable status for more than six months, and 46.9% of those patients had their condition worsen or even progress to death. In order to figure out key factors associated with different clinical outcomes, the authors summarized and compared the clinical profiles, shown in Table 2. Patients in the treatment withdrawal or stabilization groups had lower rates of ILD or CK levels. In contrast, in the aggravation or death group, most patients combined with ILD or RP-ILD, with elevated CK levels, approximately 42.1% of the patients were anti-MDA5 positive and 64.5% were anti-MAAs positive.

**Table 2:** Clinical features of DM patients with different clinical outcomes.

Variables	Treatment withdrawal or stabilization	Aggravation or Death	p-value
	n = 86	n = 76	
General information			
Female, n (%)	63 (73.3)	58 (76.3)	0.790
Age (>50 years), n (%)	43 (50.0)	43 (56.6)	0.497
Disease course, median (range), months	6.5 (3, 17.5)	6 (2, 24)	0.547
Follow-up, median (range), months	18.5 (11.2, 38.8)	13 (1, 38)	**0.031**
Clinical manifestations			
Skin			
Skin ulcer, n (%)	14 (16.3)	13 (17.1)	1.000
Heliotrope rash, n (%)	53 (61.6)	47 (61.8)	1.000
Gottron papule, n (%)	57 (66.3)	47 (61.8)	0.672
Mechanic’s hand, n (%)	5 (5.81)	7 (9.21)	0.601
Muscle & Joint			
Myalgia, n (%)	20 (23.3)	14 (18.4)	0.575
Muscle weakness, n (%)	58 (67.4)	38 (50.0)	0.036
Arthritis, n (%)	6 (7.0)	12 (15.8)	0.126
Arthralgia, n (%)	32 (37.2)	27 (35.5)	0.953
Lung			
ILD, n (%)	73 (84.9)	73 (96.1)	**0.035**
RP-ILD, n (%)	0 (0.0)	27 (35.5)	**<0.001**
Elevated CK levels, n (%)	32 (37.2)	52 (68.4)	**<0.001**
Increased ESR or CRP levels, n (%)	35 (40.7)	31 (40.8)	1.000
Auto-antibody			
MSAs, n (%)	48 (55.8)	40 (52.6)	0.804
Anti-Mi2, n (%)	6 (7.0)	2 (2.6)	0.284
Anti-TIF1γ, n (%)	10 (11.6)	2 (2.6)	0.060
Anti-MDA5, n (%)	29 (33.7)	32 (42.1)	0.349
Anti-NXP2, n (%)	3 (3.5)	2 (2.6)	1.000
Anti-SAE, n (%)	0 (0.0)	2 (2.6)	0.219
Anti-Jo1, n (%)	4 (4.7)	7 (9.2)	0.402
Anti-PL7, n (%)	3 (3.5)	5 (6.6)	0.476
Anti-PL12, n (%)	3 (3.5)	0 (0.0)	0.248
Anti-EJ, n (%)	2 (2.3)	0 (0.0)	0.499
Anti-OJ, n (%)	0 (0.0)	0 (0.0)	‒
MAAs	39 (45.3)	49 (64.5)	**0.023**
All negative, n (%)	17 (19.8)	11 (14.5)	0.496

Data were given as median (range), or as number and percentage. Elevated CK level were defined as >200 U/L. Increased ESR level were defined as >20 mm/h (female) or >15 mm/h (male). Increased CRP level were defined as >10 mg/L.

ESR, Erythrocyte Sedimentation Rate, CRP, C-Reactive Protein; ILD, Interstitial Lung Disease; CK, Creatine Kinase, MSAs, Myositis-Specific Antoantibodies; Mi2, Complex Nucleosome Remodeling Histone Deacetylase; TIF1γ, Transcription Intermediary Factor-1γ; MDA5, Melanoma Differentiation Associated protein 5; NXP2, Nuclear Matrix Protein-2; SAE, SUMO-Activating Enzyme subunit SAE; Jo1, Histidyl-ARN-t-synthetase; PL7, Threonine-ARN-tsynthetase, PL12, Alanine-ARN-tsynthetase, EJ, Glycyl-ARN-t-synthetase; OJ, Isoleucyl-ARN-t-synthetase; MAAs, Myositis-Associated autoantibodies.

Bold font for p ≤ 0.05.

## Discussion

In this study, unsupervised hierarchical cluster analysis identified three clusters among 162 DM patients based on epidemiologic, serologic, and clinical data, with patients showing significant differences in clinical manifestation, laboratory tests and clinical outcomes across clusters.

There were significant differences in myositis antibody profiles between the three clusters, the major autoantibodies in clusters 1, 2 and 3 were ARS, anti-MDA5, and anti-Mi2/anti-TIF1γ, respectively. In cluster 1, clear clinical associations were found between anti-ARS antibodies and increased risk of ILD or lower rates of RP-ILD. As in cluster 2, similar to those of previous myositis cohort studies in other countries,^9^, ^10^ MDA5^+^ RP-ILD patients with a typical dermatomyositis-specific rash and higher mortality were grouped in one cluster, suggesting that heliotrope rash, Gottron papule, ILD and anti-MDA5 antibodies are key risk factors for poor prognosis in DM. TIF1γ is a 155 kDa nuclear protein discovered by Targoff et al. by immunoprecipitation,^11^ and most cohort research indicated that anti-TIF1γ is highly associated with an increased risk of malignancy in myositis.^12^, ^13^, ^14^ In this cohort study, anti-TIF1γ was considered more common in mild DM (cluster 3). The authors further analyzed the clinical phenotype of anti-TIF1γ positive patients and found that those patients presented with a lower rate of ILD, arthritis, arthralgia, myalgia, skin ulcer, mechanic’s hand or high rate of cutaneous findings such as heliotrope rash and Gottron papule. These results were partly similar to another 134 cases of myositis cohort at Stanford University.^15^ As for anti-Mi2, it has been recognized that patients with positive anti-Mi2 antibodies have better responses and prognosis to treatment.^16^ These indicated that the algorithm accurately classified antibodies associated with better prognosis (anti-TIF1γ and anti-Mi2) into the mild DM cluster, which provided further support for clinical studies to classify patients based on their serology.

We artificially classified patients by different clinical outcomes such as withdrawal, stabilization, aggravation or death, the proportion of aggravation or death, or mortality rate increased across the three clusters, indicating that the cross-sectional data analysis after treatment was able to divide patients into three states: low, middle and high risk of poor prognosis (aggravation or death), corresponding to the cluster 3, cluster 1 and cluster 2, respectively. Actually, the International Myositis Assessment & Clinical Studies Group (IMACS) developed and validated standardized measures to assess disease activity and outcomes, known as core set measures, mainly used in clinical trials.^17^, ^18^ However, due to the large number of items in these scales, it is difficult for clinicians to complete the measures within the limited time available for outpatient consultations. In this case, unsupervised classification algorithms based on clinical serological testing could be a promising tool to provide convenient outcome measures in patients.

Limitations of this study include: 1) The number of cases is limited for the subsequent construction and validation of predictive models. The results would be more representative if multi-center cohort data were available. 2) As a retrospective cohort study, the authors were not able to review all skin symptoms or pathological changes in detail or to further assess lesions. Keeping more detailed records of physical signs would provide more information for clinical analysis.

## Conclusion

In summary, an unsupervised hierarchical cluster analysis of 162 cases with DM identified three distinct clusters with different risks of poor outcomes based on diverse clinical profiles. Hierarchical cluster analysis based on clinical manifestations and serological tests has the potential to distinguish between different phenotypes. The new clinical subgroups analyzed in this way provide us with an important reference to further explore the occurrence and development of the disease and contribute to the implementation of individualized treatment and improved patient outcomes.

## Financial support

Funding for the study was supported by the National Natural Science Foundation of China, China (nº 81771765).

## Authors’ contributions

Ting Huang: Conception and planning of the study; obtaining and interpretation of the data; critical review of the literature; statistical analysis; elaboration and writing of the manuscript; approval of the final version of the manuscript.

Ting Ding: Data collection; Effective participation in the research guidance; approval of the final version of the manuscript.

Liqing Ding: Data collection; approval of the final version of the manuscript.

Shasha Xie: Data collection; approval of the final version of the manuscript.

Xiaojing Li: Data collection; approval of the final version of the manuscript.

Qiming Meng: Data collection; approval of the final version of the manuscript.

Xiaomeng Wu: Data collection; approval of the final version of the manuscript.

Hui Luo: Conception and planning of the study; critical review of important intellectual content; approval of the final version of the manuscript.

Hongjun Zhao: Conception and planning of the study; effective participation in the research guidance; critical review of important intellectual content; approval of the final version of the manuscript.

## Conflicts of interest

None declared.
