# Supplementary material for: A new proposal for phenotypic classification and outcome assessment of dermatomyositis based on clinical manifestations and serological testing
Source: An Bras Dermatol. 2024 Mar 23;99(3):342–9. doi: 10.1016/j.abd.2023.06.005 (PMC11074555; doi:10.1016/j.abd.2023.06.005)
Supplement: Supplementary file 1 [file mmc1.doc]

ABD-D-23-00140_Supplementary Material

**Table Supplementary 1** ANOVA post-hoc tests of differences in demographics and clinical characteristics for three clusters in Table 1.

| **Variables** | **Cluster 1 vs. 2** | **Cluster 1 vs. 3** | **Cluster 2 vs. 3** |
| --- | --- | --- | --- |
|  | **p-value** | **p-value** | **p-value** |
| General information |  |  |  |
| Female, n (%) | 0.267 | 1.000 | 0.252 |
| Age (>50 years), n (%) | 1.000 | 1.000 | 1.000 |
| Disease course, median (range), months | 0.702 | 1.000 | 0.231 |
| Follow-up, median (range), months | 1.000 | 1.000 | 1.000 |
| Clinical manifestations |  |  |  |
| Skin |  |  |  |
| Skin ulcer, n (%) | 1.000 | 1.000 | 1.000 |
| Heliotrope rash, n (%) | 0.003 | 0.003 | 0.078 |
| Gottron papule, n (%) | 0.003 | 0.003 | 0.840 |
| Mechanic’s hand, n (%) | 1.000 | 1.000 | 1.000 |
| Muscle & Joint |  |  |  |
| Myalgia, n (%) | 0.003 | 0.048 | 0.867 |
| Muscle weakness, n (%) | 0.042 | 1.000 | 0.042 |
| Arthritis, n (%) | 1.000 | 1.000 | 1.000 |
| Arthralgia, n (%) | 0.504 | 0.804 | 0.015 |
| Lung |  |  |  |
| ILD, n (%) | 0.267 | 0.003 | 0.003 |
| RP-ILD, n (%) | 0.003 | 0.231 | 0.255 |
| Elevated CK levels, n (%) | 0.003 | 0.084 | 0.003 |
| Increased ESR or CRP levels, n (%) | 1.000 | 0.345 | 0.843 |
| Auto - antibody |  |  |  |
| MSAs, n (%) | 0.129 | 1.000 | 0.066 |
| Anti-Mi2, n (%) | 0.564 | 0.876 | 0.036 |
| Anti-TIF1γ, n (%) | 1.000 | 0.003 | 0.003 |
| Anti-MDA5, n (%) | 0.009 | 0.003 | 0.003 |
| Anti-NXP2, n (%) | 1.000 | 1.000 | 1.000 |
| Anti-SAE, n (%) | 1.000 | 1.000 | 1.000 |
| Anti-Jo1, n (%) | 0.003 | 0.003 | . |
| Anti-PL7, n (%) | 1.000 | 0.321 | 0.645 |
| Anti-PL12, n (%) | 1.000 | 1.000 | 1.000 |
| Anti-EJ, n (%) | 1.000 | 1.000 | 1.000 |
| Anti-OJ, n (%) | . | . | . |
| MAAs | 0.030 | 0.003 | 0.003 |
| All negative, n (%) | 1.000 | 0.003 | 0.003 |

Data were given as median (range), or as number and percentage. Elevated CK level were defined as >200 U/L. Increased ESR level were defined as >20 mm/h (female) or >15 mm/h (male). Increased CRP level were defined as >10 mg/L.

ESR, Erythrocyte Sedimentation Rate; CRP, C-Reactive Protein; ILD, Interstitial Lung Disease; RP-ILD, Rapidly Progressive Interstitial Lung Disease; CK, Creatine Kinase; MSAs, Myositis-specific antoantibodies; Mi2, Complex nucleosome remodeling histone deacetylase; TIF1γ, Transcription Intermediary Factor-1γ; MDA5, Melanoma Differentiation Associated protein 5; NXP2, Nuclear Matrix Protein-2; SAE, SUMO-activating enzyme subunit SAE; Jo1, Histidyl-ARN-t-synthetase; PL7, Threonine-ARN-tsynthetase; PL12, Alanine-ARN-tsynthetase; EJ, glycyl-ARN-t-synthetase; OJ, Isoleucyl-ARN-t-synthetase; MAAs, Myositis-Associated Autoantibodies.

For post-hoc tests, Bonferroni method was used for p-value adjustment.
